# Auswirkung der COVID-19-Pandemie auf die Versorgung von Schwerverletzten: Analyse aus dem TraumaRegister DGU®

**DOI:** 10.1007/s00113-023-01325-w

**Published:** 2023-06-21

**Authors:** Patrick Pflüger, Rolf Lefering, Michael Dommasch, Peter Biberthaler, Karl-Georg Kanz

**Affiliations:** 1 Klinik und Poliklinik für Unfallchirurgie, Klinikum rechts der Isar, Technische Universität München, München, Deutschland; 2 Institut für Forschung in der Operativen Medizin (IFOM), Universität Witten/Herdecke, Witten, Deutschland; 3 Fakultät für Medizin, Zentrale Interdisziplinäre Notaufnahme, Klinikum rechts der Isar, Technische Universität München, München, Deutschland

**Keywords:** SARS-CoV-2, Schockraum, Notfallversorgung, Unfall, Intensivstation, SARS-CoV-2, Emergency room, Emergency patient care, Trauma, Intensive care unit

## Abstract

**Hintergrund:**

Die Behandlung von Schwerverletzten bedarf intensivmedizinischer Kapazitäten, welche insbesondere während der COVID-19-Pandemie eine entscheidende Ressource darstellten. Das Ziel dieser Studie war es deshalb, die Auswirkung auf die Versorgung von Schwerverletzten unter Berücksichtigung der intensivmedizinischen Behandlung COVID-19-positiver Patienten zu analysieren.

**Methoden:**

Demografische, präklinische und intensivmedizinische Behandlungsdaten aus dem TraumaRegister DGU® der Deutschen Gesellschaft für Unfallchirurgie (DGU) der Jahre 2019 und 2020 wurden analysiert. Eingeschlossen wurden nur Schwerverletzte aus dem Bundesland Bayern. Die stationären Behandlungsdaten der COVID-19-Patienten in Bayern im Jahr 2020 wurden mittels IVENA eHealth ermittelt.

**Ergebnisse:**

Im Untersuchungszeitraum wurden 8307 Schwerverletzte im Bundesland Bayern behandelt. Insgesamt zeigte sich kein Rückgang der Anzahl der Schwerverletzten im Jahr 2020 (*n* = 4032) im Vergleich zu 2019 (*n* = 4275) (*p* = 0,4). Hinsichtlich der COVID-19-Fallzahlen wurden in den Monaten April und Dezember mit täglich über 800 Patienten auf einer Intensivstation Maximalwerte erreicht. In der kritischen Phase (≥ 100 COVID-19-Patienten auf Intensivstation) zeigte sich eine verlängerte Rettungszeit (64,8 ± 32,5 vs. 67,4 ± 30,6 min; *p* = 0,003). Die Verweildauer und die Behandlung von Schwerverletzten auf einer Intensivstation wurden nicht durch die COVID-19-Pandemie negativ beeinflusst.

**Diskussion:**

Die intensivmedizinische Versorgung von Schwerverletzten konnte während der kritischen Phasen der COVID-19-Pandemie gewährleistet werden. Die verlängerten präklinischen Rettungszeiten zeigen mögliches Optimierungspotenzial der horizontalen Integration von Präklinik und Klinik auf.

## Abstract (en)

**Background:**

The treatment of major trauma patients requires intensive care capacity, which is a critical resource particularly during the coronavirus disease 2019 (COVID-19) pandemic. Therefore, the aim of this study was to analyze the impact on major trauma care considering the intensive care treatment of COVID-19 positive patients.

**Methods:**

Demographic, prehospital, and intensive care treatment data from the TraumaRegister DGU® of the German Trauma Society (DGU) in 2019 and 2020 were analyzed. Only major trauma patients from the state of Bavaria were included. Inpatient treatment data of COVID-19 patients in Bavaria in 2020 were obtained using IVENA eHealth.

**Results:**

In total, 8307 major trauma patients were treated in the state of Bavaria in the time period investigated. The number of patients in 2020 (*n* = 4032) compared to 2019 (*n* = 4275) was not significantly decreased (*p* = 0.4). Regarding COVID-19 case numbers, maximum values were reached in the months of April and December with more than 800 intensive care unit (ICU) patients per day. In the critical period (> 100 patients with COVID-19 on ICU), a prolonged rescue time was evident (64.8 ± 32.5 vs. 67.4 ± 30.6 min; *p* = 0.003). The length of stay and ICU treatment of major trauma patients were not negatively affected by the COVID-19 pandemic.

**Conclusion:**

The intensive medical care of major trauma patients could be ensured during the high-incidence phases of the COVID-19 pandemic. The prolonged prehospital rescue times show possible optimization potential of the horizontal integration of prehospital and hospital.

## Hintergrund und Fragestellung

Das neuartige Severe Acute Respiratory Syndrome Coronavirus 2 (SARS-CoV-2/Schweres-akutes-Atemwegssyndrom-Coronavirus Typ 2) hat 2020 eine Pandemie ausgelöst, mit inzwischen über 500 Mio. Infizierten und mehr als 6 Mio. Toten (Stand: Ende 2022) [13]. Zahlreiche Maßnahmen, bis hin zum globalen „Lockdown“, wurden zur Kontrolle der Coronavirus-Disease-2019(COVID-19)-Pandemie erlassen. Diese führten zu weitreichenden Einschränkungen des öffentlichen Lebens und Ressourcenreallokation im Gesundheitssektor, um Kapazitäten für die Behandlung von Patienten mit einer SARS-CoV-2-Infektion bereitzustellen [2]. Dies beinhaltete u. a. auch die Absage von elektiven operativen Eingriffen, um insbesondere intensivmedizinische Kapazitäten zu schaffen [2].

Eine Umfrage von 72 als TraumaZentrum DGU zertifizierten Kliniken im Jahr 2020 zeigte, dass aufgrund der Ressourcenreallokation in den Krankenhäusern während des Lockdowns nur etwa ein Fünftel der normalen Operationskapazitäten zur Verfügung stand [17].

Die Einschränkungen des öffentlichen Lebens bedingten einen deutlichen Rückgang an verunfallten Patienten, insbesondere infolge von Verkehrsunfällen, Sportverletzungen und Unfällen im Freien [5, 21, 22]. Bei der Prävalenz von hüftgelenknahen Verletzungen oder Schwerverletzten zeigten sich jedoch keine wesentlichen Veränderungen [6, 9, 19]. Neben den wenigen Unfallpatienten, welche von einer SARS-CoV‑2 Infektion unmittelbar betroffen waren, wirkten sich gerade die Krankenhausmaßnahmen und Umverteilungen von Intensivkapazitäten auf die Patientenbehandlung aus [21]. Inwiefern die Behandlung von COVID-19-positiven Patienten auf einer Intensivstation die Versorgung von Schwerverletzten beeinflusst hat, ist jedoch nicht genauer untersucht.

In Bayern wurde aufgrund der Allgemeinverfügung vom 19.03.2020 ein einheitliches, IT-gestütztes System zur Erfassung der Behandlungskapazitäten und COVID-19-Patienten eingeführt [11]. Die Krankenhäuser wurden verpflichtet, die täglichen Fallzahlen verbindlich und fortlaufend elektronisch zu übermitteln [3, 7]. Hierdurch war eine tagesaktuelle und somit reale Abbildung des Infektionsgeschehens und vor allem der Behandlungskapazitäten der Krankenhäuser möglich [3].

Das Ziel dieser Studie war es deshalb, die Auswirkung auf die Versorgung von Schwerverletzten unter Berücksichtigung der intensivmedizinischen Behandlung COVID-19-positiver Patienten zu analysieren.

## Methoden

Es handelt sich um eine retrospektive Studie mit Daten aus dem TraumaRegister DGU® der Deutschen Gesellschaft für Unfallchirurgie (DGU) und dem IT-System IVENA eHealth. Die TraumaRegister DGU®(TR-DGU)-Daten von 2019 und 2020 sowie die IVENA-eHealth-COVID-Sonderlage-Daten für das Bundesland Bayern von 2020 wurden analysiert. Der Einschlusszeitraum für die IVENA-eHealth-COVID-Daten umfasste den Zeitraum vom 19.03.2020 bis einschließlich 31.12.2020. Einschlusskriterien für die TR-DGU-Daten waren:

Primär versorgt in einer bayerischen Klinik (Zuverlegungen ausgeschlossen); Basisdatensatz (AIS mind. 3; AIS 2, nur falls intensivmedizinisch behandelt).

Die vorliegende Arbeit steht in Übereinstimmung mit der Publikationsrichtlinie des TraumaRegister DGU® und ist registriert unter der TR-DGU-Projekt-ID 2020-050.

### TraumaRegister DGU®

Das TR-DGU wurde 1993 gegründet. Ziel dieser multizentrischen Datenbank ist eine pseudonymisierte und standardisierte Dokumentation von Schwerverletzten.

Die Daten werden prospektiv in 4 aufeinanderfolgenden Phasen gesammelt: A) präklinische Phase, B) Schockraum und anschließende OP-Phase, C) Intensivstation und D) Entlassung. Die Dokumentation beinhaltet detaillierte Informationen über Demografie, Verletzungsmuster, Komorbiditäten, präklinisches und klinisches Management, intensivmedizinischen Verlauf, wichtige Laborbefunde, einschließlich Transfusionsdaten, sowie das Outcome. Das Einschlusskriterium ist die Aufnahme in das Krankenhaus über den Schockraum mit anschließender Intensiv- oder Intermediate-Care-Überwachung oder Ankunft in der Klinik mit Vitalzeichen und Versterben vor Aufnahme auf die Intensivstation.

Die Infrastruktur für Dokumentation, Datenmanagement und Datenanalyse wird von der AUC – Akademie der Unfallchirurgie GmbH, welche der DGU angegliedert ist, bereitgestellt. Die wissenschaftliche Führung liegt bei der Sektion Notfall‑, Intensivmedizin und Schwerverletztenversorgung (Sektion NIS) der DGU. Über eine webbasierte Anwendung geben die teilnehmenden Kliniken ihre Daten pseudonymisiert in eine zentrale Datenbank ein. Wissenschaftliche Auswertungen werden nach einem in der Publikationsrichtlinie des TR-DGU festgeschriebenen Peer-Review-Verfahren genehmigt.

Die teilnehmenden Kliniken sind primär in Deutschland (90 %) lokalisiert, aber eine zunehmende Anzahl von Kliniken aus anderen Ländern trägt ebenfalls Daten bei (zurzeit aus Österreich, Belgien, China, Finnland, Luxemburg, Slowenien, Schweiz, den Niederlanden und den Vereinigten Arabische Emiraten). Derzeit fließen jährlich über 35.000 Fälle aus fast 700 Kliniken in die Datenbank ein. Die Beteiligung am TR-DGU ist freiwillig, für die dem TR-DGU zugehörigen Kliniken ist die Eingabe zumindest eines Basisdatensatzes zur Qualitätssicherung verpflichtend.

Die Prognose der Sterblichkeit der Patienten wurde mittels Revised Injury Severity Classification (RISC) II Score berechnet und mit der beobachteten Mortalität verglichen [12].

### IVENA eHealth

Seit Februar 2013 werden in Bayern die Krankenhauszuweisungen durch die Rettungsleitstelle mittels IT-System (IVENA eHealth [IVENA], interdisziplinärer Versorgungsnachweis, Fa. mainis IT-Service GmbH, Offenbach am Main, Deutschland) disponiert. Aufgrund der Allgemeinverfügung vom 19.03.2020 und Erweiterung vom 24.03.2020 wurden bayernweit die Krankenhäuser verpflichtet, die Fallzahlen und Krankenhausbelegungen über das IT-Programm IVENA verbindlich und fortlaufend zu dokumentieren [7, 11]. Von den 478 bayerischen Kliniken, die im IVENA-Sonderlagen-Modul hinterlegt sind, waren 292 Kliniken an der Behandlung von COVID-19-Patienten beteiligt. Es wurden 2 Phasen anhand der durchschnittlichen Anzahl an COVID-19-positiven Patienten im Jahr 2020 auf Intensivstationen definiert:

- ≥ 100 COVID-positive Patienten pro Tag = „kritische Phase“,
- < 100 COVID-positive Patienten pro Tag = „unkritische Phase“.

Die so definierten Zeiträume des Jahres 2020 wurden entsprechend auf das Jahr 2019 für den Vergleich übertragen.

### Statistische Auswertung

Häufigkeiten wurden mit Anzahl und Prozent berechnet, metrische Daten mit Mittelwert und Standardabweichung (SD). Beim Vergleich von metrischen Daten in 2 Gruppen wurde ein *t*-Test durchgeführt oder der nichtparametrische Mann-Whitney-U-Test im Falle deutlicher Abweichung von einer Normalverteilung. Im Fall von kategorialen Variablen wurde der Exakte-Fisher-Test verwendet. Ein *p*-Wert < 0,05 wurde als statistisch signifikant gewertet. Die Analyse der Daten erfolgte mittels SPSS (IBM SPSS Statistics for Windows, Version 26.0, IBM Corp., Armonk, NY, USA).

## Ergebnisse

**Figure 1:**
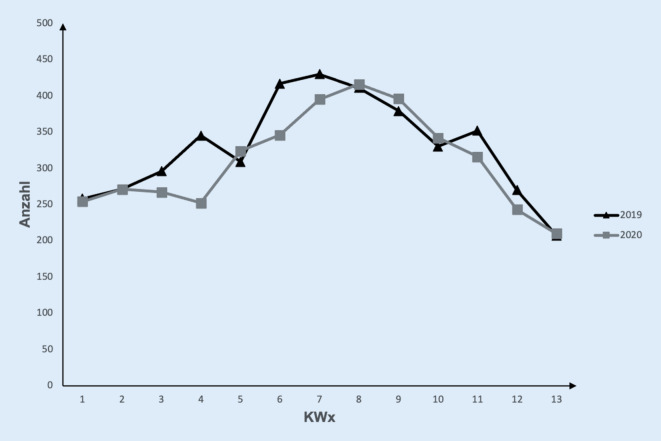

Im Untersuchungszeitraum wurden insgesamt 8307 Schwerverletzte im Bundesland Bayern behandelt. Die Patientenkohorten von 2019 und 2020 haben sich hinsichtlich des Alters und der Verletzungsschwere nicht signifikant unterschieden (Tab. 1). Betrachtet man den Unfallhergang, so zeigten sich im Lockdownjahr 2020 im Vergleich zum Vorjahr etwas weniger Motorrad- und Autounfälle (Tab. 1). Fahrradunfälle waren häufiger zu beobachten und Stürze blieben relativ konstant (Tab. 1). Insgesamt zeigte sich kein Rückgang der Anzahl der Schwerverletzten im Jahr 2020 im Vergleich zu 2019 (*p* = 0,4). Lediglich für den Zeitraum der Kalenderwochen 13–16 war ein geringgradiger Rückgang der Patientenzahlen zu beobachten (Abb. 1). Von insgesamt 2260 im Schockraum durchgeführten COVID-19-Tests im Jahr 2020 waren lediglich 22 Patienten positiv auf SARS-CoV‑2 (1 %) getestet worden.

**Table 1:** 

	2019	2020
**Anzahl**	4275	4032
**Alter**	53,6 (SD 22,0)	55,0 (SD 22,3)
**ISS**	17,5 (SD 11,5)	17,9 (SD 11,1)
**Unfallhergang**		
*Auto*		
Anzahl	921	695
Aufnahme ins Krankenhaus	21,7 %	17,4 %
*Motorrad*		
Anzahl	601	487
Aufnahme ins Krankenhaus	14,2 %	12,2 %
*Fahrrad*		
Anzahl	493	576
Aufnahme ins Krankenhaus	11,6 %	14,4 %
*Fußgänger*		
Anzahl	191	138
Aufnahme ins Krankenhaus	4,5 %	3,5 %
*Sturz aus großer Höhe (>* *3* *m)*		
Anzahl	586	615
Aufnahme ins Krankenhaus	13,8 %	15,4 %
*Sturz aus niedriger Höhe*		
Anzahl	1039	1099
Aufnahme ins Krankenhaus	24,5 %	27,5 %
*Sonstige*		
Anzahl	409	390
Aufnahme ins Krankenhaus	9,6 %	9,8 %

**Figure 2:**
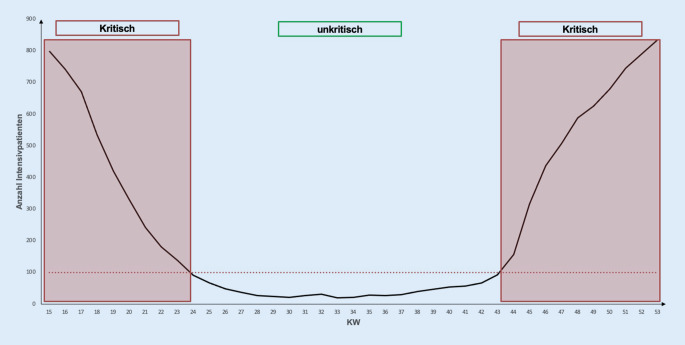

Die Anzahl der COVID-19-positiven Patienten, welche in Bayern auf Intensivstationen behandelt wurden, ist in Abb. 2 dargestellt. Entsprechend wurden KW 14–23 und KW 44–53 als „kritische Phasen“ (≥ 100 COVID-positive Patienten/Tag) definiert (Abb. 2). Im Mittel wurden 275 Patienten/Tag auf einer Intensivstation aufgrund einer Infektion mit SARS-CoV‑2 behandelt. Die Maximalwerte wurden in den Monaten April und Dezember mit täglich über 800 COVID-19-positiven Patienten erreicht. Die niedrigsten Werte waren im Juli und August mit weniger als 30 Patienten pro Tag zu beobachten.

Betrachtet man die Rettungszeit in der kritischen und unkritischen Phase, so zeigte sich eine verlängerte Rettungszeit in der kritischen Phase 2020 (*p* = 0.003, Tab. 2). Hinsichtlich der Verweildauer auf der Intensivstation haben sich keine Unterschiede zwischen den Jahren 2019 und 2020 gezeigt (Tab. 3). Auch bei der Anzahl und dem Anteil der Schwerverletzen mit intensivmedizinischer Behandlung waren zwischen der kritischen und unkritischen Phase 2020 sowie im Vergleich zu 2019 keine signifikanten Veränderungen zu beobachten (Tab. 4).

**Table 2:** 

	Zeit	Anzahl
Unkritische Phase	64,8 (SD 32,5)	1405
Kritische Phase	67,4 (SD 30,6)*	1010

*p = 0,003

**Table 3:** 

	Verweildauer in Tagen Mittelwert/Median (IQR)	Anzahl
*2019*		
Unkritisch	4,6/2 (1–5)	1924
Kritisch	4,4/2 (1–4)	1442
*2020*		
Unkritisch	4,7/2 (1–5)	1876
Kritisch	4,8/2 (1–5)	1318

*IQR* Interquartilbereich

**Table 4:** 

	Intensivbehandlung *n*, (%)	
Phase	2019	2020
Unkritisch	1641 (85,3)	1552 (82,7)
Kritisch	1203 (83,4)	1104 (83,8)

Definition „unkritisch“ und „kritisch“ anhand der Anzahl der COVID-19-Patienten auf einer Intensivstation im Jahr 2020

*n* Gesamtzahl, *%* Prozent aller Schockraumpatienten

Im Jahr 2019 sind von 3858 Patienten 374 im Krankenhaus verstorben (9,7 %) und 2020 311 von 3562 Patienten (8,7 %). Es zeigte sich kein signifikanter Unterschied zwischen der beobachteten und erwarteten (RISC II) Mortalität für die untersuchten Jahre (Tab. 5).

**Table 5:** 

	2019	2020
Anzahl der Patienten	3858	3562
Mortalität im Krankenhaus	374 (9,7 %)	311 (8,7 %)
Prognose, basierend auf RISC II	9,0 %	9,0 %

## Diskussion

In dieser Studie konnte mittels Analyse des TraumaRegister DGU® und der COVID-19-Belegungsdaten für das Bundesland Bayern gezeigt werden, dass die intensivmedizinische Behandlung COVID-19-positiver Patienten keinen wesentlichen Einfluss auf die Versorgung von Schwerverletzten hatte. Lediglich die präklinische Rettungszeit war in der kritischen Phase 2020 im Vergleich zum Vorjahr verlängert.

Insgesamt haben wir keinen Rückgang der Anzahl an Schwerverletzten im Jahr 2020 im Vergleich zu 2019 festgestellt. Die erste Lockdownphase 2020 bedingte nur einen kurzfristigen Einbruch der Patientenzahlen. Eine Übersichtsarbeit mit internationalen Studien zeigte hingegen, dass die mit der COVID-19-Pandemie verbundenen Maßnahmen insgesamt zu einem Rückgang von verunfallten Patienten geführt hatte [21]. In die Analyse wurden alle Patienten eingeschlossen, welche aufgrund eines Unfalls behandelt wurden. Der Rückgang der unfallbedingten Verletzungen war insbesondere bei Freizeitaktivitäten und Verkehrsunfällen zu beobachten [21]. Betrachtet man hingegen vornehmlich die schwer verletzten Patienten, so zeigten sich in Deutschland und den Niederlanden relativ konstante Zahlen [4, 5, 9]. Insbesondere die Versorgung von diesen Patienten bedarf intensivmedizinischer Kapazitäten, welche während der COVID-19-Pandemie eine entscheidende Ressource darstellten [3, 4].

Eine landesweite Studie in den Niederlanden konnte sogar feststellen, dass aufgrund der COVID-19-Pandemie weniger schwer verletzte Patienten auf einer Intensivstation behandelt wurden und die Mortalität im Vergleich zu einer ähnlichen Patientenkohorte der Jahre 2018/2019 signifikant höher war [4]. Die Autoren führen diese Beobachtung auf mögliche Engpässe von Intensivkapazitäten zurück, welche durch die Behandlung von COVID-19-erkrankten Patienten entstanden sind. In unserer Studie zeigte sich für das Bundesland Bayern hingegen kein geringerer Anteil an Schwerverletzten mit intensivmedizinischer Behandlung oder eine verkürzte Verweildauer auf der Intensivstation. Dies lässt sich trotz hoher COVID-19-Fallzahlen im Bundesland möglicherweise auf die gesundheitspolitischen Maßnahmen zurückführen [3, 11]. So wurden landesweit u. a. elektive operative Eingriffe abgesagt, um insbesondere intensivmedizinische Behandlungskapazitäten zu schaffen [2]. Dies hatte zu weitreichenden Folgen sowohl für die Gesundheitsdienstleister als auch insbesondere Patienten geführt [10, 18]. So wirkten sich die Maßnahmen auch negativ auf das Überleben von Patienten mit Krebserkrankungen aus [15].

Die beobachteten Veränderungen hinsichtlich des Unfallhergangs waren im Jahr 2020 vergleichbar mit anderen Studien, welche den Einfluss der COVID-19-Pandemie auf Traumapatienten untersucht haben [21]. Auto‑/Motorradunfälle sind seltener ursächlich gewesen und Fahrradunfälle sowie Stürze aus dem Stand („low fall“) traten anteilig häufiger auf [1, 5, 19]. Gerade aufgrund der konstant hohen Zahl an Niedrigenergietraumen als Ursache für die unfallchirurgische Behandlung stellten andere Studien ein höheres Durchschnittsalter bei verunfallten Patienten während der COVID-19-Lockdownphase fest [1, 5, 8, 19]. Das mittlere Patientenalter im Jahr 2020 war nicht signifikant höher als 2019, was auf die Einschlusskriterien des untersuchten Kollektivs im Vergleich zu den anderen Studien zurückzuführen ist. Die Verletzungsschwere hat sich nicht zwischen den analysierten Patientenkollektiven von 2019 und 2020 unterschieden. Ebenso zeigte sich die prognostizierte Überlebenswahrscheinlichkeit und beobachtete Mortalität nahezu unverändert. Dies hat sich so auch in einer Übersichtsarbeit gezeigt, welche internationale Studien zum Einfluss der COVID-19-Pandemie untersucht hatte, und festgestellt hat, dass sich der ISS bei den meisten Studien nicht wesentlich verändert hatte [21].

Die präklinische Rettungszeit war in den kritischen Phasen des Jahres 2020 im Vergleich zum Vorjahr verlängert. Dies konnte so auch in einer Studie in den Niederlanden beobachtet werden [5]. Mögliche Erklärungen für die verlängerten Rettungszeiten sind die aufwendigeren persönlichen Schutzmaßnahmen im Rahmen der COVID-19-Pandemie und die vermehrte Abmeldung von Krankenhäusern an der Akutversorgung aufgrund von fehlenden Kapazitäten [14, 16]. Der zunehmende Anstieg von Zwangsbelegungen von Notaufnahmen war bereits vor der COVID-19-Pandemie zu beobachten und verschärfte sich in der Pandemiesituation aufgrund fehlender Behandlungskapazitäten [16]. Da eine verlängerte präklinische Rettungszeit das Überleben von Traumapatienten negativ beeinflussen kann, gilt es zukünftig, die Koordination im Rettungswesen weiter zu optimieren [20]. Darüber hinaus müssen entsprechende Behandlungskapazitäten geschaffen werden, um die Notfallversorgung sowie die Weiterbehandlung von allen Patienten weiterhin gewährleisten zu können.

### Limitationen

Die vorliegende Studie weist aufgrund des retrospektiven Designs Limitationen auf, welche bei der Interpretation der Daten zu beachten sind. Es wurden nur die Daten des TR-DGU für das Bundesland Bayern analysiert, und daher können die Ergebnisse nicht uneingeschränkt auf ganz Deutschland bezogen werden. Darüber hinaus war aufgrund der Pseudonymisierung der TR-DGU-Daten nur eine Zuordnung zum Bundesland möglich. Für die Auswertung wurden deshalb die COVID-Sonderlagen-Daten der einzelnen Kliniken über IVENA eHealth zusammengeführt.

## Schlussfolgerungen

- Die intensivmedizinische Versorgung von Schwerverletzten konnte während der kritischen Phase der COVID-19-Pandemie gewährleistet werden.
- Die Anzahl und Verletzungsschwere der Schwerverletzten wurden nicht wesentlich durch die COVID-19-Restriktionen beeinflusst.
- Während der kritischen Phasen der COVID-19-Pandemie haben sich längere präklinische Rettungszeiten gezeigt.
- Eine verbesserte horizontale Integration von Präklinik und Klinik bietet die Chance, die Behandlungskapazitäten in den kritischen Phasen einer Pandemie weiter zu optimieren.
